# Associations between Dietary Inflammatory Index, ultra-processed food intake, and clinical outcomes in women with lipedema

**DOI:** 10.3389/fnut.2026.1846293

**Published:** 2026-06-30

**Authors:** Nurgul Arslan

**Affiliations:** Department of Nutrition and Dietetics, Atatürk Faculty of Health Sciences, Dicle University, Diyarbakir, Türkiye

**Keywords:** dietary inflammatory index, inflammation, lipedema, Mediterranean diet, ultra-processed foods

## Abstract

**Objectives:**

To examine the associations of ultra-processed food (UPF) consumption, dietary inflammatory index (DII), and Mediterranean diet adherence with pain severity, physical quality of life, body composition, and inflammatory markers in women with lipedema.

**Methods:**

This cross-sectional study included women diagnosed with lipedema across different disease stages. Dietary intake was assessed using a validated food frequency questionnaire, and foods were classified according to the NOVA system to determine UPF consumption. The dietary inflammatory index was calculated to assess the inflammatory potential of the diet, and Mediterranean diet adherence was evaluated using a standardized scoring system. Anthropometric measurements, body composition parameters, inflammatory markers, pain intensity (VAS), and physical quality of life (SF-12 PCS) were assessed. Multivariable regression analyses were performed to investigate the associations between dietary variables and clinical outcomes.

**Results:**

A total of 86 women with lipedema (stage 1: *n* = 36, stage 2: *n* = 33, stage 3: *n* = 17) were included. UPF consumption increased from 28.1 to 41.3% of total energy and DII scores from +1.46 to +3.02 across stages, while Mediterranean diet adherence decreased from 28.2 to 21.3. In parallel, BMI increased from 27.1 to 31.1 kg/m^2^ and body fat percentage from 36.7 to 41.1%. Inflammatory markers also rose across stages (hs-CRP: 3.9 to 6.1 mg/L; IL-6: 3.1 to 4.6 pg/ml). In multivariable models, higher DII scores were associated with increased pain severity (β = 0.29, *p* = 0.007) and higher hs-CRP levels (β = 0.41, *p* < 0.001), whereas Mediterranean diet adherence was positively associated with physical quality of life (β = 0.34, *p* = 0.002).

**Conclusion:**

Higher ultra-processed food consumption and dietary inflammatory potential were associated with increased inflammation, pain, and adiposity, whereas greater Mediterranean diet adherence was associated with better physical quality of life in womendietary inflammatory index, inflammation, lipedema, Mediterranean diet, ultra-processed foods with lipedema.

## Introduction

Lipedema is a chronic, progressive adipose tissue disorder characterized by pain, tenderness, easy bruising, and disproportionate fat accumulation in the lower extremities (1). The condition occurs predominantly in women and is associated with hormonal periods such as puberty, pregnancy, and menopause. Although the pathophysiology of lipedema has not been fully elucidated, microvascular dysfunction, inflammation in subcutaneous adipose tissue, lymphatic insufficiency, and alterations in connective tissue are reported to play key roles (2). Recent study has demonstrated that lipedema is not merely a cosmetic problem, but rather a systemic condition leading to chronic pain, mobility limitations, and a marked reduction in quality of life (3).

Growing evidence suggests that the inflammatory burden in lipedema is higher than previously assumed and plays a decisive role in pain severity, edema level, and functional capacity (4). Local inflammation in adipose tissue, macrophage infiltration, extracellular matrix irregularities, and fibrosis are among the pathological features of lipedema; these processes also contribute to elevations in systemic inflammatory markers (5). Therefore, lifestyle factors that modulate inflammation particularly dietary patterns are considered a critical target in the management of lipedema.

Dietary habits play a central role in the regulation of inflammation, and in recent years, studies examining the relationship between dietary patterns and lipedema have begun to increase (6). Ultra-processed foods (UPFs), as defined by the NOVA classification system, are industrial formulations made mostly or entirely from substances derived from foods (e.g., oils, fats, sugars, starches, and protein isolates) and typically contain additives such as flavor enhancers, colorings, emulsifiers, and preservatives, with little or no intact whole food content (7). UPF intake has been shown in various populations to influence pain perception, increase inflammatory markers, and adversely affect body fat distribution, particularly in women (8). However, no study to date has investigated the relationship between UPF consumption and clinical manifestations in women with lipedema, indicating a substantial gap in the existing literature.

The Dietary Inflammatory Index (DII) is a validated, internationally recognized scoring system developed to assess the inflammatory potential of the diet. Higher DII scores have been associated with inflammatory diseases, metabolic disorders, and chronic pain (9). Considering that inflammation is a prominent component of lipedema, evaluating the relationship of DII with pain, quality of life, and anthropometric parameters in this population is of considerable clinical importance (10). Recent evidence suggests that dietary interventions with lower Dietary Inflammatory Index (DII) scores, particularly those rich in anti-inflammatory and antioxidant components, may contribute to reductions in systemic inflammatory markers such as CRP and IL-6 in women with lipedema (3).

One of the anti-inflammatory dietary models, the Mediterranean Diet, emphasizes the consumption of olive oil, vegetables, fruits, whole grains, fish, and legumes, and its chronic inflammation–reducing effects have been demonstrated in numerous studies (11, 12). The Mediterranean Diet has been shown in various populations to confer beneficial effects on pain management, adipose tissue distribution, and metabolic health (13). Although evidence on the assessment of Mediterranean Diet adherence in women with lipedema is limited, available findings suggest that this dietary pattern may improve quality of life and reduce inflammatory markers (14).

Given the metabolic and inflammatory burden of lipedema, this study aimed to examine the associations of ultra-processed food consumption, dietary inflammatory potential, and Mediterranean diet adherence with pain, quality of life, body composition, and inflammatory markers in women with lipedema.

## Materials and methods

### Study design and population

This research is an analytical cross-sectional study designed to examine the relationships between dietary patterns and pain, body composition, inflammatory markers, and quality of life in women with lipedema presenting to the Nutrition Outpatient Clinic of Malatya Turgut Özal Training and Research Hospital. The study was conducted between January 2024 and June 2025. Eligible patients with a diagnosis of lipedema who presented to the outpatient clinic were consecutively evaluated, and individuals meeting the inclusion and exclusion criteria were enrolled after obtaining written informed consent. The study population consisted of women aged 18–45 years with a confirmed diagnosis of lipedema who presented to the relevant outpatient clinic during the specified period.

#### Ethical approval

The study protocol was reviewed and approved by the Clinical Research Ethics Committee of Malatya Turgut Özal University (Approval No: 2024/3, Date: 01/9/2024). The research was conducted in accordance with the principles of the Declaration of Helsinki. All participants were thoroughly informed about the aim of the study, data collection procedures, and potential benefits and risks. Participation was voluntary, and written informed consent was obtained from those who agreed to take part.

#### Sample and sample size

The study sample consisted of 86 women with lipedema who met the inclusion criteria. Initially, 102 individuals were assessed; 16 were excluded from the analysis due to not meeting the inclusion criteria, having incomplete datasets, or providing unreliable dietary intake records. The sample size was calculated using G^*^Power software, taking into account the estimated number of independent variables to be used in multivariable regression and structural equation modeling (SEM) analyses. Assuming a medium effect size (f^2^= 0.15), 80% power, and a 5% type I error, a minimum of 80 participants was deemed sufficient. Therefore, the final sample size of 86 participants was considered statistically adequate for both regression analyses and SEM (15).

#### Inclusion and exclusion criteria

Only women aged 18–45 years with a clinically confirmed diagnosis of lipedema were included in the study. The inclusion criteria were defined as the presence of lipedema findings for at least 6 months, regular follow-up visits to the outpatient clinic, stable medical treatment within the last 3 months, and sufficient cognitive capacity to complete sociodemographic forms and dietary intake records. Exclusion criteria included pregnancy or lactation, active malignancy or major systemic infection, advanced renal or hepatic failure, severe psychiatric disorders, history of bariatric surgery, use of systemic corticosteroids or immunosuppressive therapy, and participation in a ketogenic or highly restrictive diet program within the last 3 months.

#### Diagnosis and staging of lipedema

The diagnosis of lipedema was established by two specialist physicians experienced in lipedema management from the Physical Medicine and Rehabilitation and Vascular Surgery departments of the hospital. Diagnostic criteria were based on those defined in international lipedema consensus statements (16, 17). Key diagnostic features included symmetrical fat accumulation in the lower extremities, painful or tender nodular adipose tissue on palpation, easy bruising, a sensation of increasing edema toward the end of the day, and relative sparing of the feet. Detailed medical history and physical examination were performed to assess symptom onset, disease progression, association with hormonal periods, and previous treatment attempts. Lipedema stage was classified as stage 1, 2, or 3 according to clinical findings and cutaneous/subcutaneous changes, and stage information for each participant was recorded in the data collection form (18).

#### Data collection procedure

Data collection was conducted through face-to-face interviews by a trained dietitian at the nutrition outpatient clinic. During the initial visit, sociodemographic data, educational and employment status, household income, smoking and alcohol use, physical activity habits, and sedentary time were obtained using a standardized questionnaire. In the same session, information on lipedema-related symptoms (disease duration, leg tenderness, easy bruising, increased edema at the end of the day, presence of daily pain, functional limitations) and existing chronic conditions (hypothyroidism, hypertension, PCOS, etc.) was collected in detail. On the same day, anthropometric measurements were taken, bioelectrical impedance analysis (BIA) was performed, participants were instructed on how to complete dietary intake records, and pain and quality of life scales were administered. Fasting blood samples were obtained in the hospital biochemistry laboratory in the morning after a 10–12-h overnight fast, following standardized protocols.

#### Sociodemographic and lifestyle variables

Sociodemographic variables included age, marital status, educational level, employment status, and household income level. Lifestyle variables comprised smoking status (yes/no), alcohol consumption (consumption ≥ once per month: yes/no), physical activity level, daily step count, sleep duration, and daily sedentary time. Physical activity level was categorized as low, moderate, or high based on weekly duration of moderate-to-vigorous physical activity. Participants' average daily step counts during the previous week were recorded via smartphone applications or pedometer data. Sleep duration and sedentary time were evaluated based on self-reported average daily hours.

#### Dietary assessment and calculation of dietary patterns

To determine individuals' dietary intake, both a quantitative food frequency questionnaire (FFQ) and a non-consecutive 3-day 24-h dietary recall (two weekdays and one weekend day) were utilized. The quantities of consumed foods and portion sizes of meals were estimated using the Food and Meal Photograph Catalog and the Standardized Recipes for Institutional Catering (19, 20). Dietary data were analyzed using the Nutrition Information System (BeBIS) software, enabling the calculation of daily energy and nutrient intakes (21).

The quantitative FFQ assessed the frequency and amount of consumption over the past 3 months across 13 food groups, including milk and dairy products; meat, meat products, and eggs; legumes; bread and cereals; vegetables; fruits; oilseeds; fats; beverages; alcoholic beverages; sweets and miscellaneous foods; fast food; spices; and others. To facilitate recall, participants were asked how often and how much they consumed these foods. Consumption frequency was categorized into eight levels and the average daily intake of each food item was calculated by multiplying the reported consumption amount by the corresponding coefficient.

### Evaluation of the Dietary Inflammatory Index (DII) and mediterranean diet adherence

The Dietary Inflammatory Index (DII), developed by Shivappa et al. (22), is a population-based tool used to assess the inflammatory potential of diets across different populations. The index includes 45 dietary parameters, such as energy, macronutrients, fatty acids (including saturated, monounsaturated, and polyunsaturated fatty acids, omega-3 and omega-6 fatty acids), cholesterol, trans fats, carbohydrates, fiber, alcohol, vitamins (A, β-carotene, D, E, B-complex, C), minerals (iron, magnesium, zinc, selenium), and various bioactive compounds (e.g., flavonoids, caffeine, and certain spices).

For each dietary parameter available in the dataset, an inflammatory effect score was calculated based on global reference values (mean and standard deviation), and these scores were summed to obtain the individual's overall DII score. Due to the absence of certain components (β-carotene, flavan-3-ols, flavones, flavonols, flavanones, anthocyanidins, isoflavones, and eugenol) in the BeBIS database, these were excluded from the calculation, and a total of 37 parameters were used in this study. There is no established cut-off point for categorizing DII scores as low, moderate, or high. Higher scores indicate a pro-inflammatory diet, whereas lower scores reflect an anti-inflammatory dietary pattern. The DII score was calculated based on data obtained from the non-consecutive 3-day dietary records (23).

Mediterranean Diet (MD) adherence was evaluated using the 14-item MedDietScore, which assesses 12 dietary habits and 2 MD-related practices. Each criterion met scores 1 point, yielding a total score between 0 and 14, where higher scores indicate greater adherence. The Turkish version of the tool has been validated (24–26).

### Assessment of ultra-processed food (UPF) consumption

Dietary intake was assessed using a semi-quantitative food frequency questionnaire (FFQ) validated for the Turkish population (27). Participants were asked to report their habitual consumption frequency of foods and beverages over the previous year using predefined categories (“never”, “every day”, “1–2 times/week”, “3–4 times/week”, “5–6 times/week”, “1–2 times/month”). Reported frequencies were converted into daily intake values (g/day). Portion sizes were estimated using a standardized Food and Meal Photo Catalog to improve accuracy (19).

Food items were classified according to the NOVA classification system, which categorizes foods based on the extent and purpose of industrial processing. Ultra-processed foods (UPF) were identified and further grouped into predefined subcategories based on previous literature (7, 28). The proportion of total energy intake derived from UPF (% energy) was calculated for each participant. For categorical analyses, participants were classified according to the median value of UPF consumption, consistent with previous studies and to facilitate comparisons in relatively small sample sizes (29, 30). Each item was assigned to one of the following categories: unprocessed/minimally processed, processed, or ultra-processed, and the percentage of total energy intake derived from ultra-processed foods (UPF % energy) was calculated (31). The detailed classification of food items according to the NOVA system is provided in Supplementary File 1.

#### Anthropometric measurements and body composition

Anthropometric measurements were obtained by the same team according to standardized protocols. Height was measured without shoes, in an upright position, using a stadiometer. Body weight was measured in light clothing using a calibrated digital scale. Body mass index (BMI) was calculated as weight in kilograms divided by height in meters squared. Waist circumference was measured at the midpoint between the lowest costal margin and the iliac crest along the midaxillary line; hip circumference was measured at the level of the greater trochanter using a non-stretch measuring tape. The waist-to-hip ratio was calculated by dividing waist circumference by hip circumference.

Body composition was assessed using a multifrequency bioelectrical impedance analysis device (InBody 770, InBody Co., Seoul, Korea). Before measurement, participants were instructed to fast for at least 3 h, avoid caffeine and strenuous physical activity, and empty their bladder. Total body fat percentage, fat-free mass, lower-extremity segmental fat percentage, and visceral fat score were recorded using BIA. To evaluate the regional distribution of lipedema, thigh and calf circumferences of both lower extremities were measured at standardized anatomical landmarks, and mean values were calculated.

#### Biochemical measurements

Fasting blood samples were drawn from the antecubital vein after a 10–12-h overnight fast and analyzed in the hospital biochemistry laboratory in accordance with institutional protocols. Serum glucose, total cholesterol, HDL-cholesterol, LDL-cholesterol, and triglyceride levels were measured using enzymatic colorimetric methods, and insulin levels were measured using an immunochemiluminescence assay. Insulin resistance was calculated using the HOMA-IR formula, and HOMA-IR≥2.5 was considered indicative of prediabetes/insulin resistance (32).

Inflammatory markers included high-sensitivity C-reactive protein (hs-CRP), interleukin-6 (IL-6), and tumor necrosis factor-alpha (TNF-α). hs-CRP levels were measured using a high-sensitivity nephelometric method, while IL-6 and TNF-α levels were analyzed using commercial ELISA kits. IL-6 and TNF-α levels were analyzed using commercially available ELISA kits (e.g., Elabscience, Wuhan, China) according to the manufacturer's instructions. Absorbance was measured using a microplate reader (e.g., BioTek ELx800, USA).All biochemical measurements were performed in accordance with the laboratory's internal and external quality control procedures.

#### Assessment of pain and quality of life

Pain intensity was assessed using a 10-cm Visual Analog Scale (VAS) to evaluate the average pain severity in lipedema-affected regions. Participants were asked to mark the intensity of their pain over the past week on a line ranging from 0 (no pain) to 10 (unbearable pain). The mean VAS score was recorded as a continuous variable. A cut-off value of VAS ≥ 6 was used to define the high-pain group in logistic regression analyses (33).

Health-related quality of life was assessed using the physical component summary (PCS) of the SF-12 questionnaire to evaluate the physical dimension. SF-12 PCS scores were calculated using a standardized algorithm, with higher scores indicating better physical quality of life. In the analyses, physical quality of life was used as a continuous variable (34).

#### Other clinical and psychosocial variables

Clinical variables related to lipedema symptoms included disease duration, stage, leg tenderness, easy bruising, increased edema at the end of the day, presence of daily pain, and movement limitations. Comorbid conditions (hypothyroidism, PCOS, hypertension, vitamin D deficiency, pre-diabetes) and previously diagnosed depression and/or anxiety were recorded based on participant self-report and/or verification from medical records. Use of nonsteroidal anti-inflammatory drugs (NSAIDs) or analgesics for pain management was also assessed.

#### Statistical analysis

Statistical analyses were performed using IBM SPSS Statistics for Windows, version 26.0 (IBM Corp., Armonk, NY, USA) and an additional SEM-compatible software package (e.g., AMOS, LISREL, or R/lavaan) for structural equation modeling (35). The distribution of continuous variables was assessed using the Kolmogorov–Smirnov and Shapiro–Wilk tests. Variables that met the assumption of normality were summarized as mean ± standard deviation, whereas non-normally distributed variables were presented as median (interquartile range). Categorical variables were expressed as numbers and percentages.

One-way analysis of variance (ANOVA) or the appropriate nonparametric alternative was used to compare anthropometric, nutritional, and biochemical variables across lipedema stages. When significant differences were observed, suitable *post hoc* tests were applied for multiple comparisons. Relationships between dietary patterns and anthropometric, inflammatory, and clinical variables were examined using Pearson or Spearman correlation analyses, depending on variable distribution, and the results were presented as a correlation matrix.

Prior to multivariable analysis, bivariate analyses were performed to assess the association between independent variables and outcome variables. Variables with a *p*-value < 0.20 were considered for inclusion in the multivariable models to avoid excluding potentially important predictors. Additionally, variables with established clinical relevance based on previous literature were included in the models regardless of their statistical significance. This approach is consistent with established recommendations for variable selection in regression modeling (36, 37). Multiple linear regression analyses were conducted to identify independent predictors of pain (VAS), hs-CRP, and physical quality of life (SF-12 PCS).

Multiple linear regression analyses were conducted to identify independent predictors of pain (VAS), hs-CRP, and physical quality of life (SF-12 PCS). In these models, UPF % energy, DII score, and MedDietScore were entered as the main dietary variables, while BMI and age were included as covariates. Three separate models were constructed, with VAS score, hs-CRP level, and SF-12 PCS score as dependent variables, respectively. Regression assumptions (normality, linearity, multicollinearity, and homoscedasticity of residuals) were evaluated using residual plots and variance inflation factor (VIF) values; models were reconsidered if VIF >10.

Although analyses were stratified by lipedema stage, stage was not included as a covariate in the regression models to avoid potential overadjustment and multicollinearity, given its close relationship with anthropometric and inflammatory variables. Additionally, the relatively limited sample size restricted the number of variables included in the models.

Structural equation modeling (SEM) was applied to examine the direct and indirect relationships between dietary patterns, inflammation, pain, and physical quality of life. In the model, a latent variable labeled “pro-inflammatory diet” was constructed, with UPF % energy, DII score, and reverse-coded MedDietScore loading onto this factor. A second latent variable labeled “inflammation” was defined, with hs-CRP, IL-6, and TNF-α as its indicators. In the structural part of the model, it was specified that pro-inflammatory diet predicts inflammation, inflammation predicts pain, inflammation predicts physical quality of life, and pro-inflammatory diet predicts both pain and physical quality of life. Model fit was assessed using the chi-square/degrees of freedom ratio (χ^2^/df), Comparative Fit Index (CFI), Tucker–Lewis Index (TLI), Root Mean Square Error of Approximation (RMSEA), and Standardized Root Mean Square Residual (SRMR). CFI and TLI values above 0.90 and RMSEA and SRMR values below 0.08 were considered indicative of good model fit. A two-sided *p*-value < 0.05 was accepted as the threshold for statistical significance in all analyses.

## Results

As shown in Table 1, the mean age of participants was 34.2 ± 6.1 years. The majority were university-educated (54.6%) and employed (60.4%). Low physical activity was reported in 67.4% of participants, with a mean daily step count of 5,412 ± 2,086. The mean BMI was 28.7 ± 4.2 kg/m^2^, with 39.5% classified as overweight and 36.1% as obese. Most participants were in lipedema stage 1 (41.9%) or stage 2 (38.3%). Leg tenderness (87.2%), evening edema (81.4%), and daily pain (72.1%) were commonly reported. The mean pain score was 5.8 ± 2.1. Vitamin D deficiency (47.6%) and prediabetes (22.1%) were among the most frequent comorbidities.

**Table 1:** Sociodemographic, lifestyle, anthropometric, and clinical characteristics of participants (*n* = 86).

Variable	Value
Sociodemographic characteristics	
Age (years)	34.2 ± 6.1
Marital status	
Married	39 (45.3%)
Single	47 (54.7%)
Education level	
Low (primary/secondary school)	18 (20.9%)
Moderate (high school)	21 (24.4%)
High (university or higher)	47 (54.6%)
Employment status	
Employed	52 (60.4%)
Unemployed/not working	34 (39.6%)
Monthly household income	
Low	42 (48.9%)
Middle/high	44 (51.1%)
Lifestyle characteristics	
Smoking status	
Yes	18 (20.9%)
No	68 (79.1%)
Alcohol consumption (≥1/month)	
Yes	14 (16.3%)
No	72 (83.7%)
Physical activity level	
Low	58 (67.4%)
Moderate	20 (23.2%)
High	8 (9.3%)
Average daily step count	5,412 ± 2,086 steps/day
Sleep duration (hours/day)	6.8 ± 1.2
Sedentary time (hours/day)	7.1 ± 2.4
Anthropometric and body composition	
Body weight (kg)	74.6 ± 11.2
Height (cm)	161.2 ± 6.4
Body mass index (kg/m^2^)	28.7 ± 4.2
BMI categories (WHO classification)	
Normal weight (18.5–24.9)	21 (24.4%)
Overweight (25.0–29.9)	34 (39.5%)
Obesity (≥30.0)	31 (36.1%)
Waist circumference (cm)	88.3 ± 10.4
Hip circumference (cm)	108.2 ± 9.6
Waist-to-hip ratio	0.82 ± 0.06
Body fat percentage (%)	38.4 ± 5.8
Lower-limb fat percentage (%)	41.9 ± 6.4
Thigh circumference (cm)	60.8 ± 7.3
Calf circumference (cm)	38.6 ± 4.5
Lipedema-related clinical findings	
Lipedema stage	
Stage 1	36 (41.9%)
Stage 2	33 (38.3%)
Stage 3	17 (19.8%)
Duration of lipedema (years)	8.4 ± 4.7
Family history of lipedema	
Yes	29 (33.7%)
No	57 (66.3%)
Leg tenderness	75 (87.2%)
Easy bruising	64 (74.4%)
Evening edema increase	70 (81.4%)
Daily pain presence	62 (72.1%)
Pain severity (VAS, 0–10)	5.8 ± 2.1
Mobility limitation(Yes)	49 (57.0%)
Use of NSAIDs/analgesics	21 (24.4%)
Comorbidities	
Hypothyroidism	15 (17.4%)
Polycystic ovary syndrome (PCOS)	12 (13.9%)
Vitamin D deficiency	41 (47.6%)
Hypertension	8 (9.3%)
Prediabetes (HOMA-IR ≥ 2.5)	19 (22.1%)
Depression/anxiety diagnosis	23 (26.7%)

Data are presented as mean ± standard deviation (SD) for continuous variables and number (percentage) for categorical variables.

BMI categories were defined according to World Health Organization (WHO) criteria: normal weight (18.5–24.9 kg/m^2^), overweight (25.0–29.9 kg/m^2^), and obesity (≥30.0 kg/m^2^).

Physical activity level was categorized as low, moderate, or high based on self-reported weekly activity duration.

Prediabetes was defined as HOMA-IR ≥ 2.5.

BMI, Body Mass Index; VAS, visual analog scale; PCOS, polycystic ovary syndrome; HOMA-IR, homeostatic model assessment of insulin resistance; NSAID, non-steroidal anti-inflammatory drug.

As shown in Table 2, total energy intake and macronutrient distribution did not differ significantly across lipedema stages (*p* > 0.05). In contrast, saturated fat intake increased from 24.5 ± 7.1 g/day in stage 1 to 29.8 ± 9.3 g/day in stage 3 (*p* = 0.04). Dietary fiber intake decreased (22.4 ± 6.8 to 16.8 ± 5.7 g/day, *p* = 0.003), whereas added sugar intake increased (26.3 ± 11.4 to 34.5 ± 13.2 g/day, *p* = 0.04). Similarly, omega-3 (1.21 ± 0.46 to 0.89 ± 0.39 g/day, *p* = 0.02) and vitamin D intake (4.3 ± 1.9 to 3.2 ± 1.4 μg/day, *p* = 0.03) decreased across stages. UPF consumption increased both as a percentage of total energy (28.1 ± 7.9 to 41.3 ± 9.1%) and as servings per day (2.1 ± 0.9 to 3.6 ± 1.4) (*p* < 0.001). DII scores increased from +1.46 ± 1.12 to +3.02 ± 1.41 (*p* < 0.001), while MedDietScore decreased from 28.2 ± 4.7 to 21.3 ± 4.8 (*p* < 0.001). Olive oil consumption decreased from 5.8 ± 2.1 to 3.4 ± 1.8 times/week, and fruit and vegetable intake decreased from 4.3 ± 1.1 to 2.9 ± 1.2 portions/day (*p* ≤ 0.002). In contrast, red and processed meat intake increased from 2.1 ± 1.0 to 3.6 ± 1.6 servings/week (*p* = 0.002).

**Table 2:** Dietary intake, diet quality, and inflammatory profile across lipedema stages.

Variable	Stage 1 (*n* = 36)	Stage 2 (*n* = 33)	Stage 3 (*n* = 17)	*p*-value
Energy and macronutrient intake				
Total energy (kcal/day)	1,782 ± 298	1,866 ± 314	1,943 ± 355	0.09
Carbohydrate (%)	45.1 ± 5.8	47.2 ± 6.5	48.3 ± 6.8	0.18
Protein (%)	18.6 ± 2.9	17.4 ± 3.0	16.8 ± 3.2	0.12
Total fat (%)	36.3 ± 5.1	35.1 ± 5.3	34.2 ± 5.9	0.22
Saturated fat (g/day)	24.5 ± 7.1	27.2 ± 8.4	29.8 ± 9.3	0.04^*^
Diet quality and key nutrients				
Dietary fiber (g/day)	22.4 ± 6.8	19.1 ± 6.2	16.8 ± 5.7	0.003^**^
Added sugars (g/day)	26.3 ± 11.4	31.8 ± 12.6	34.5 ± 13.2	0.04^*^
Omega-3 intake (g/day)	1.21 ± 0.46	1.03 ± 0.41	0.89 ± 0.39	0.02^*^
Vitamin D intake (μg/day)	4.3 ± 1.9	3.6 ± 1.6	3.2 ± 1.4	0.03^*^
Ultra-processed food (UPF) consumption				
UPF (% of total energy)	28.1 ± 7.9	33.6 ± 8.4	41.3 ± 9.1	< 0.001^***^
UPF (servings/day)	2.1 ± 0.9	2.8 ± 1.1	3.6 ± 1.4	< 0.001^***^
Dietary Inflammatory Index (DII)
DII score	+1.46 ± 1.12	+2.29 ± 1.31	+3.02 ± 1.41	< 0.001^***^
DII tertiles				
Low	18 (50.0%)	7 (21.2%)	1 (5.8%)	—
Moderate	12 (33.3%)	13 (39.4%)	4 (23.5%)	—
High	6 (16.7%)	13 (39.4%)	12 (70.6%)	—
Mediterranean diet adherence				
MedDietScore	28.2 ± 4.7	25.1 ± 4.9	21.3 ± 4.8	< 0.001^***^
High adherence (≥30)	14 (38.9%)	4 (12.1%)	1 (5.8%)	—
Olive oil consumption (times/week)	5.8 ± 2.1	4.6 ± 1.9	3.4 ± 1.8	0.002^**^
Fruit and vegetable intake (portions/day)	4.3 ± 1.1	3.6 ± 1.0	2.9 ± 1.2	< 0.001^***^
Red/processed meat intake (servings/week)	2.1 ± 1.0	2.9 ± 1.3	3.6 ± 1.6	0.002^**^

Data are presented as mean ± standard deviation (SD) for continuous variables and number (percentage) for categorical variables.

Comparisons between lipedema stages were performed using one-way ANOVA or the appropriate non-parametric equivalent, depending on data distribution. *Post hoc* analyses were conducted for significant results.

^*^p < 0.05, ^**^p < 0.01, ^***^p < 0.001 indicate statistically significant differences between groups.

UPF (% of total energy) represents the proportion of daily energy intake derived from ultra-processed foods.

DII score reflects the inflammatory potential of the diet, with higher values indicating a more pro-inflammatory diet.

MedDietScore reflects adherence to the Mediterranean diet, with higher scores indicating greater adherence.

Olive oil consumption represents the frequency of use as the primary added fat in meals.

UPF, ultra-processed food; DII, Dietary Inflammatory Index; SD, standard deviation.

As shown in Table 3, BMI increased from 27.1 ± 3.4 to 31.1 ± 4.3 kg/m^2^ across lipedema stages (*p* = 0.004). Waist circumference (84.9 ± 8.6 to 94.2 ± 10.7 cm, *p* = 0.003) and hip circumference (105.1 ± 8.9 to 112.7 ± 10.3 cm, *p* = 0.01) also increased. Body fat percentage (36.7 ± 5.1 to 41.1 ± 6.0%, *p* = 0.01), lower-limb fat percentage (39.3 ± 5.7 to 45.2 ± 6.7%, *p* = 0.001), and visceral fat score (8.6 ± 3.1 to 11.9 ± 3.9, *p* = 0.006) showed significant increases. Inflammatory markers increased across stages, including hs-CRP (3.9 ± 1.7 to 6.1 ± 2.4 mg/L, *p* = 0.001), IL-6 (3.1 ± 1.3 to 4.6 ± 1.8 pg/ml, *p* = 0.004), and TNF-α (7.1 ± 2.4 to 9.2 ± 3.1 pg/ml, *p* = 0.02). Fasting glucose (92.1 ± 9.8 to 99.1 ± 12.3 mg/dl, *p* = 0.04) and HOMA-IR (2.3 ± 1.0 to 3.3 ± 1.4, *p* = 0.02) increased, whereas fasting insulin and prediabetes prevalence did not differ significantly (*p* > 0.05). HDL-cholesterol decreased from 56.1 ± 11.2 to 48.9 ± 9.8 mg/dl (*p* = 0.03), while total cholesterol, LDL-cholesterol, and triglycerides showed no significant differences across stages (*p* > 0.05).

**Table 3:** Anthropometric, body composition, and biochemical parameters across lipedema stages.

Variable	Stage 1 (*n* = 36)	Stage 2 (*n* = 33)	Stage 3 (*n* = 17)	*p*-value
Anthropometric parameters				
Body mass index (kg/m^2^)	27.1 ± 3.4	28.9 ± 3.8	31.1 ± 4.3	0.004^**^
Waist circumference (cm)	84.9 ± 8.6	88.7 ± 9.8	94.2 ± 10.7	0.003^**^
Hip circumference (cm)	105.1 ± 8.9	108.5 ± 9.1	112.7 ± 10.3	0.01^*^
Waist-to-hip ratio	0.81 ± 0.05	0.82 ± 0.06	0.84 ± 0.06	0.09
Body composition (BIA)				
Body fat percentage (%)	36.7 ± 5.1	38.6 ± 5.6	41.1 ± 6.0	0.01^*^
Lower-limb fat (%)	39.3 ± 5.7	42.1 ± 6.2	45.2 ± 6.7	0.001^**^
Visceral fat score	8.6 ± 3.1	10.2 ± 3.4	11.9 ± 3.9	0.006^**^
Thigh circumference (cm)	58.7 ± 6.4	61.1 ± 7.3	63.9 ± 7.9	0.03^*^
Calf circumference (cm)	37.2 ± 4.0	38.9 ± 4.4	40.4 ± 4.8	0.04^*^
Inflammatory markers				
hs-CRP (mg/L)	3.9 ± 1.7	4.8 ± 2.0	6.1 ± 2.4	0.001^**^
IL-6 (pg/ml)	3.1 ± 1.3	3.7 ± 1.5	4.6 ± 1.8	0.004^**^
TNF-α (pg/ml)	7.1 ± 2.4	8.0 ± 2.7	9.2 ± 3.1	0.02^*^
Glucose–insulin metabolism				
Fasting glucose (mg/dl)	92.1 ± 9.8	94.7 ± 10.9	99.1 ± 12.3	0.04^*^
Fasting insulin (μIU/ml)	10.6 ± 4.2	11.9 ± 4.7	13.4 ± 5.1	0.08
HOMA-IR	2.3 ± 1.0	2.7 ± 1.2	3.3 ± 1.4	0.02^*^
Prediabetes (HOMA-IR ≥ 2.5), *n* (%)	6 (16.7%)	7 (21.2%)	6 (35.3%)	0.18
Lipid profile				
Total cholesterol (mg/dl)	192.0 ± 33.4	201.3 ± 35.1	210.4 ± 37.8	0.09
HDL-cholesterol (mg/dl)	56.1 ± 11.2	52.7 ± 10.8	48.9 ± 9.8	0.03^*^
LDL-cholesterol (mg/dl)	118.2 ± 28.5	123.6 ± 29.3	132.1 ± 30.2	0.11
Triglycerides (mg/dl)	132.4 ± 47.8	141.2 ± 52.1	157.3 ± 56.4	0.12

Data are presented as mean ± standard deviation (SD) for continuous variables and number (percentage) for categorical variables.

Comparisons between lipedema stages were performed using one-way ANOVA or the appropriate non-parametric equivalent.

^*^p < 0.05, ^**^p < 0.01 indicate statistically significant differences.

BIA, bioelectrical impedance analysis; hs-CRP, high-sensitivity C-reactive protein; HOMA-IR, homeostatic model assessment of insulin resistance.

**Figure 1:**
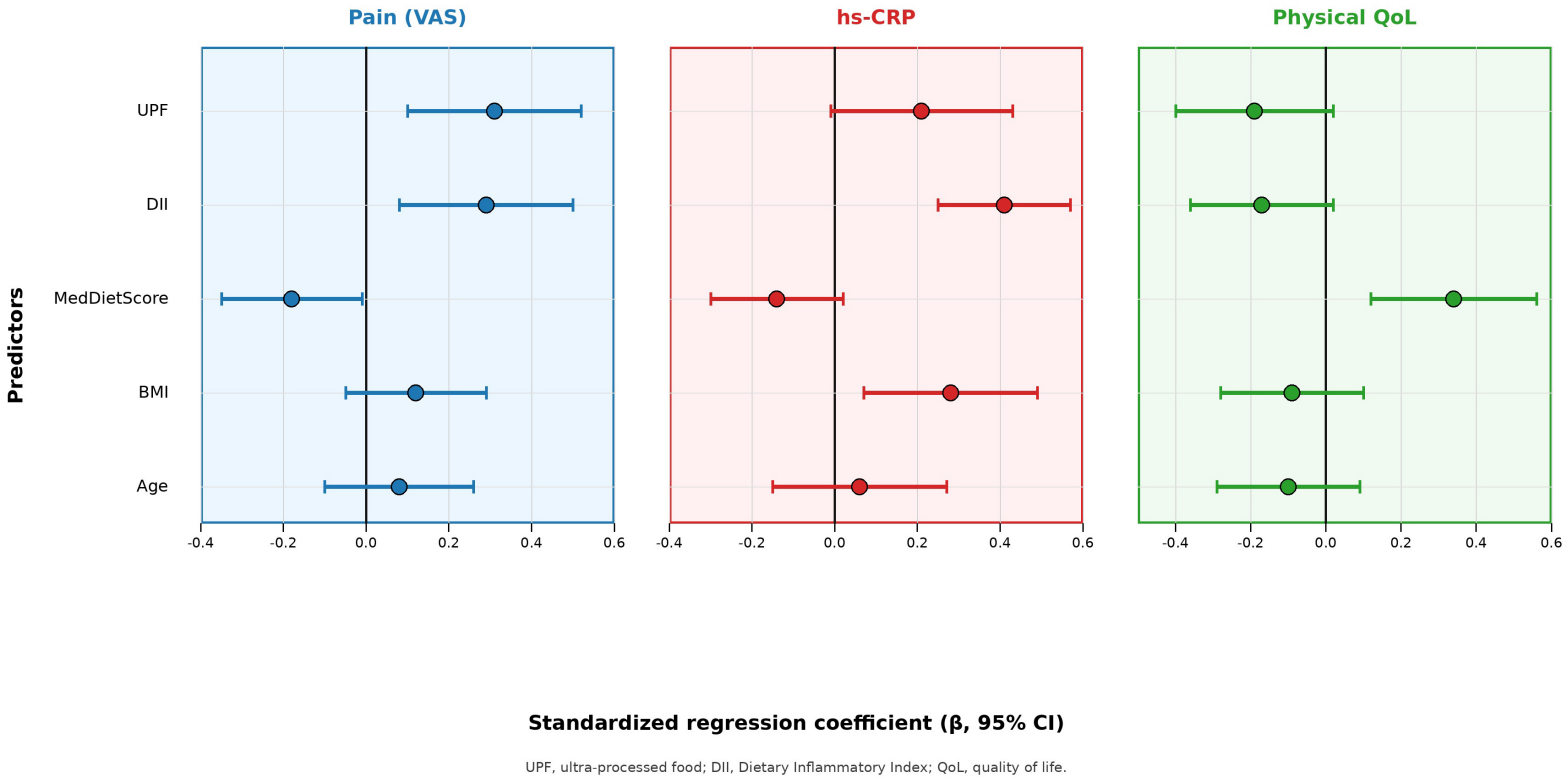
Forest plot showing standardized regression coefficients (β) and 95% confidence intervals for the associations of dietary and anthropometric variables with pain intensity (VAS), hs-CRP levels, and physical quality of life (SF-12 PCS) in women with lipedema.

**Figure 2:**
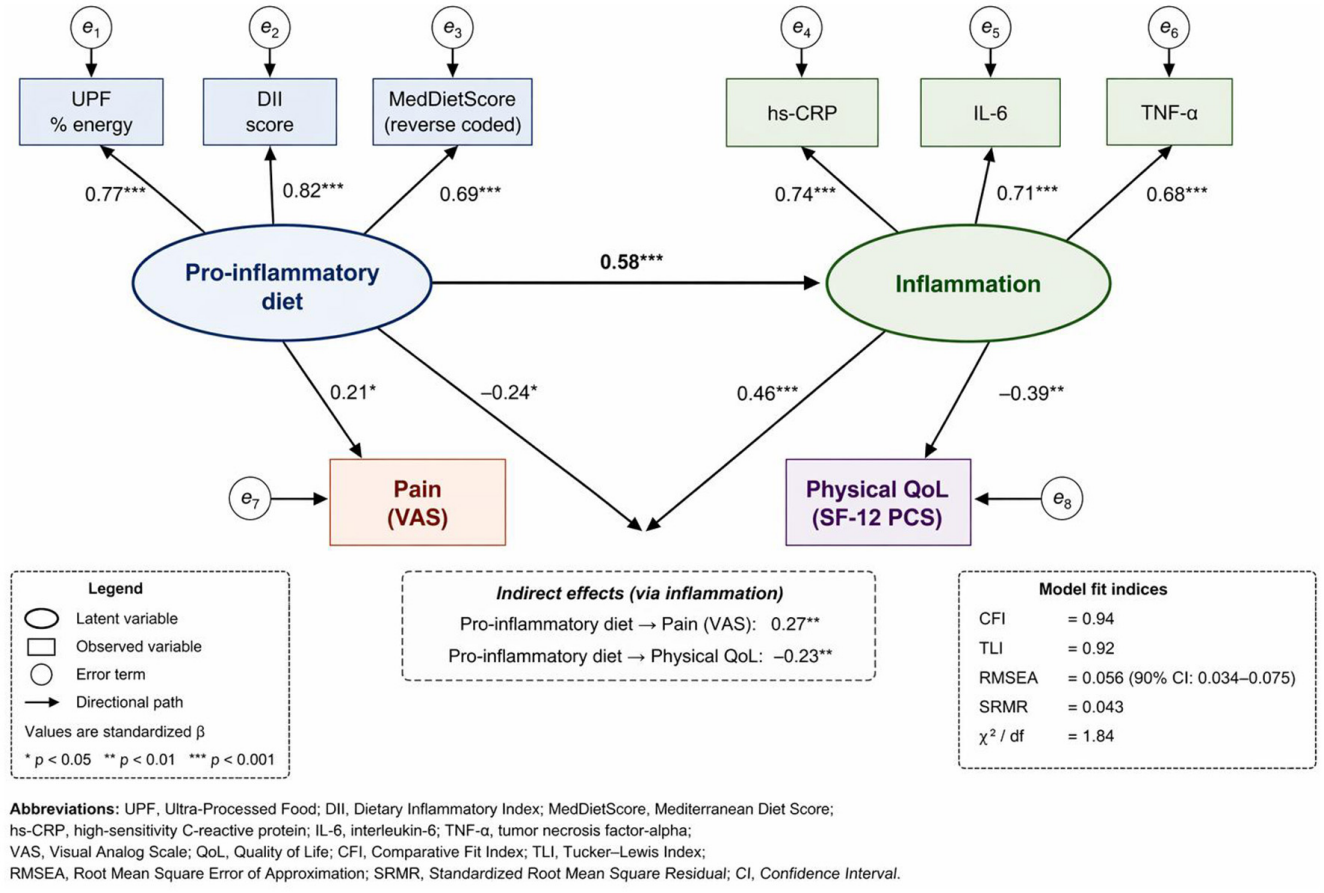
Heatmap showing correlations between dietary variables, anthropometric parameters, inflammatory markers, and clinical outcomes. Values inside the cells represent correlation coefficients (*r*). Color intensity reflects the strength and direction of correlations.

**Figure 3:**
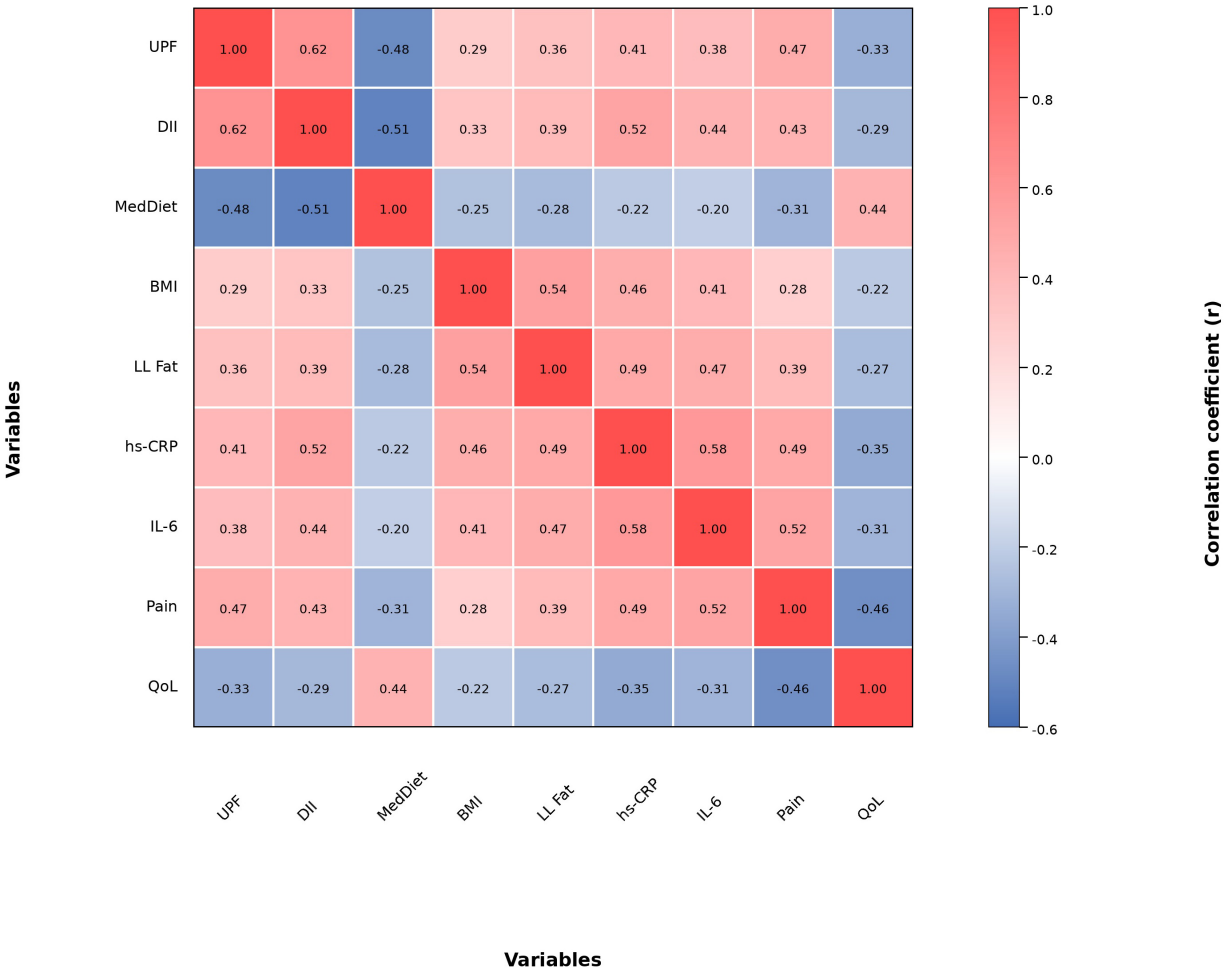
Structural equation model illustrating the relationships between dietary patterns, inflammation, pain, and physical quality of life. The latent variable “pro-inflammatory diet” was constructed from UPF % energy, DII score, and reverse-coded MedDietScore. The latent variable “inflammation” was defined by hs-CRP, IL-6, and TNF-α. Values represent standardized path coefficients (β). All displayed paths are statistically significant.

As shown in Table 4, in Model 1, UPF % energy (β = 0.31, *p* = 0.004) and DII score (β = 0.29, *p* = 0.007) were significantly associated with pain (VAS). In Model 2, DII score (β = 0.41, *p* < 0.001) and BMI (β = 0.28, *p* = 0.010) were significantly associated with hs-CRP levels. In Model 3, MedDietScore (β = 0.34, *p* = 0.002) was significantly associated with physical quality of life. Other variables were not statistically significant (*p* > 0.05). The multivariable regression findings are summarized in Figure 1. The structural equation model demonstrated the direct and indirect relationships among pro-inflammatory diet, inflammation, pain, and physical quality of life, as shown in Figures 2–3.

**Table 4:** Multivariable linear regression models for pain, inflammation, and physical quality of life.

Predictor	Model 1 pain (VAS)β (95% CI)	Model 2 hs-CRPβ (95% CI)	Model 3 physical QoLβ (95% CI)
UPF (% energy)	**0.31 (0.10 to 0.52)**	0.21 (−0.01 to 0.43)	−0.19 (−0.40 to 0.02)
DII score	**0.29 (0.08 to 0.50)**	**0.41 (0.25 to 0.57)**	−0.17 (−0.36 to 0.02)
MedDietScore	−0.18 (−0.35 to −0.01)	−0.14 (−0.30 to 0.02)	**0.34 (0.12 to 0.56)**
BMI (kg/m^2^)	0.12 (−0.05 to 0.29)	**0.28 (0.07 to 0.49)**	−0.09 (−0.28 to 0.10)
Age (years)	0.08 (−0.10 to 0.26)	0.06 (−0.15 to 0.27)	−0.10 (−0.29 to 0.09)
Model fit			
*R* ^2^	0.39	0.42	0.31
Adjusted *R*^2^	0.36	0.39	0.27
F-statistic	*F*_(5, 80)_ = 10.3^***^	*F*_(5, 80)_ = 11.8^***^	*F*_(5, 80)_ = 7.3^***^
Root MSE	1.84	1.52	4.11

β values represent standardized regression coefficients with 95% confidence intervals (CI).

Model 1: Pain (VAS) as the dependent variable. Model 2: hs-CRP as the dependent variable. Model 3: Physical quality of life (SF-12 PCS) as the dependent variable.

^*^p < 0.05, ^**^p < 0.01, ^***^p < 0.001. These symbols indicate the statistical significance levels of the model F-statistics and regression coefficients where applicable.

Bold values indicate statistically significant regression coefficients or model statistics at *p* < 0.05.

All models were adjusted for age and BMI. No multicollinearity was observed (VIF < 5).

UPF, ultra-processed food; DII, Dietary Inflammatory Index; QoL, quality of life; CI, confidence interval.

## Discussion

In this cross-sectional sample of women with lipedema, participants were predominantly in early to mid-adulthood with relatively high educational and employment levels, yet they presented with a substantial clinical burden. The coexistence of middle or higher household income with high rates of mobility limitation, daily pain, and analgesic use supports the notion that lipedema affects functioning and quality of life independently of traditional socioeconomic disadvantage.

Recent high-quality reviews and guidelines emphasize that although lipedema is not caused by obesity, excess weight, metabolic disturbances, and diet quality can exacerbate symptoms, accelerate disease progression, and worsen quality of life (38, 39). In this context, dietary strategies such as low-carbohydrate and ketogenic patterns have gained increasing attention, with intervention studies reporting improvements in body composition, pain, inflammatory markers, and quality of life (10, 40, 41). At the same time, Mediterranean-style dietary patterns are widely recommended due to their well-established anti-inflammatory and cardiometabolic benefits (11, 42). The present study demonstrated that dietary patterns differ clearly across lipedema stages, even though total energy intake remains similar. While energy and macronutrient distribution did not change significantly, important differences were observed in diet quality. Specifically, saturated fat, added sugar, and ultra-processed food (UPF) intake increased, whereas fiber, omega-3 fatty acids, and vitamin D intake decreased with advancing stage. In parallel, dietary inflammatory index (DII) scores increased and adherence to the Mediterranean diet declined. These findings suggest that diet quality, rather than calorie intake, is more closely related to disease progression.

A key finding of this study is the relationship between dietary inflammatory potential and systemic inflammation. Higher DII scores were associated with increased inflammatory markers, indicating a more pronounced inflammatory state in advanced stages. These findings are consistent with interventional evidence showing that reductions in DII are associated with decreases in inflammatory markers (3, 43). In addition, previous research in women with lipedema has demonstrated that higher DII scores are positively associated with pro-inflammatory cytokines, while adherence to a Mediterranean dietary pattern shows inverse associations with these biomarkers (43). While these studies confirm the role of diet in modulating systemic inflammation, they have not consistently demonstrated associations with clinical outcomes. In contrast, our results extend this evidence by showing that dietary inflammatory potential is also associated with symptom severity, including pain and physical quality of life.

In addition to their pro-inflammatory nutrient profile, emerging evidence suggests that the adverse health effects of ultra-processed foods may not be fully explained by their inflammatory potential alone. A large population-based study demonstrated that the association between UPF consumption and low-grade inflammation was only partially mediated by the dietary inflammatory index, indicating that other mechanisms such as food additives, industrial processing, and alterations in the food matrix may also contribute to systemic inflammation (44).

UPF consumption also increased significantly across stages in our cohort. This shift toward highly processed foods may contribute to increased inflammation and poorer clinical outcomes. The observed increase in UPF intake across disease stages is consistent with population-based findings in women, where higher consumption of ultra-processed foods has been associated with poorer physical functioning and reduced quality of life (45). (44)Together, these findings suggest that disease progression in lipedema may be accompanied by a cumulative shift toward more ultra-processed, pro-inflammatory dietary patterns, which may contribute to worsening metabolic and inflammatory profiles.

We also observed a clear decline in Mediterranean diet adherence with disease progression. Lower adherence was accompanied by reduced intake of plant-based foods and healthy fats, alongside increased consumption of animal-based and processed products. This shift toward a more pro-inflammatory dietary pattern may partly explain the worsening inflammatory and clinical profile. Intervention studies support this observation, demonstrating that Mediterranean-style dietary patterns may improve body composition and quality of life in women with lipedema (11). More broadly, adherence to Mediterranean and plant-forward dietary patterns has been consistently associated with lower levels of inflammatory markers, including CRP, IL-6, and TNF-α, as well as improved overall health status (43).

In addition to dietary patterns, our findings showed progressive increases in adiposity-related parameters across stages, including overall and lower-limb fat accumulation. These changes were accompanied by worsening dietary profiles, suggesting that diet quality may influence both inflammation and fat distribution. Although lipedema fat is known to be resistant to conventional weight loss strategies, dietary composition may still play an important role in modulating metabolic and inflammatory processes (45).

Pain and physical quality of life are central clinical features of lipedema, and our findings indicate that diet may play an important role in these outcomes. Higher dietary inflammatory potential and greater UPF intake were associated with increased pain, whereas higher adherence to the Mediterranean diet was associated with better physical quality of life. These findings are consistent with clinical intervention studies demonstrating that dietary modification can reduce pain and improve quality of life in lipedema, independent of weight loss (40). Furthermore, population-based analyses have shown that higher DII scores are associated with greater prevalence of chronic pain and poorer physical functioning, particularly among women (12). The inverse relationship between pain and physical quality of life observed in our study is also consistent with findings from both lipedema-specific and broader chronic pain populations.

The mechanisms underlying these associations are likely multifactorial. Lipedema is increasingly recognized as a condition characterized by chronic low-grade inflammation, adipose tissue dysfunction, and immune dysregulation. Emerging evidence suggests that altered immune responses to dietary antigens, intestinal permeability, and inflammatory signaling pathways may contribute to disease progression (46). In this context, pro-inflammatory dietary patterns may exacerbate these processes, whereas anti-inflammatory diets rich in fiber, omega-3 fatty acids, and antioxidants may help attenuate inflammation and improve clinical outcomes.

Importantly, our findings suggest that dietary composition may be more strongly associated with inflammation and clinical outcomes than total energy intake. This shifts the focus from calorie restriction alone to overall diet quality as a key modifiable factor. Accordingly, dietary strategies targeting inflammation may represent a promising complementary approach in the management of lipedema.

Despite these findings, lipedema is a complex condition involving structural and functional alterations in adipose tissue, and diet alone is unlikely to be sufficient as a standalone treatment. However, dietary interventions may play an important supportive role in reducing inflammation, improving metabolic health, and alleviating symptoms. These findings highlight the importance of integrating nutritional strategies into a comprehensive, multidisciplinary approach to lipedema management.

## Strengths and limitations

Some limitations should be considered. The cross-sectional design limits causal inference, and dietary intake was assessed using self-reported methods. In addition, residual confounding cannot be excluded. However, the consistency of our findings with previous studies strengthens their validity.

Future studies should adopt longitudinal or interventional designs, include larger and more diverse samples, and incorporate standardized reporting of lipedema stage, comorbidities, and nutritional treatments to strengthen causal understanding and refine tailored dietary recommendations.

## Conclusion

In conclusion, this study demonstrates that worsening diet quality—characterized by higher dietary inflammatory potential and greater consumption of ultra-processed foods, along with lower adherence to a Mediterranean dietary pattern—is associated with increased inflammation, greater pain, and poorer physical quality of life in women with lipedema. These findings support the role of diet as an important modifiable factor and highlight the potential benefits of anti-inflammatory dietary strategies as part of a comprehensive approach to lipedema management.

## Data Availability

The datasets generated and/or analyzed during the current study are not publicly available due to ethical and privacy restrictions but are available from the corresponding author upon reasonable request.
