# Supplementary material for: Associations between Dietary Inflammatory Index, ultra-processed food intake, and clinical outcomes in women with lipedema
Source: Front Nutr. 2026 Jun 30;13:1846293. doi: 10.3389/fnut.2026.1846293 (PMC13364962; doi:10.3389/fnut.2026.1846293)
Supplement: Supplementary file 1 [file Table_1.docx]

# Supplementary Table S1. Detailed NOVA Classification Used in the Study

| Category | Description and Examples |
| --- | --- |
| NOVA 1 — Unprocessed or Minimally Processed Foods | Foods in their natural state or minimally altered. Examples: whole grains, fruits, vegetables, legumes, nuts, eggs, fish, milk, plain yogurt. |
| NOVA 2 — Processed Culinary Ingredients | Substances derived from foods used in cooking. Examples: oils, butter, sugar, salt, honey, vinegar. |
| NOVA 3 — Processed Foods | Foods produced by adding salt/sugar/fat to NOVA 1. Examples: canned foods, cheese, salted nuts, traditional breads. |
| NOVA 4 — Ultra-Processed Foods (UPFs) | Industrial formulations with additives and minimal whole food. Ready-to-eat or heat products. |
| UPF Subgroup 1: Snacks and sweets | Cookies, cakes, chocolates, candies, snack bars, desserts. |
| UPF Subgroup 2: Sugar-sweetened beverages | Soft drinks, fruit drinks, energy drinks, sports drinks. |
| UPF Subgroup 3: Fast food and processed meats | Fast food meals, pizza, sausages, deli meats, bacon. |
| UPF Subgroup 4: Industrial grain products | Packaged breads, cereals, tortillas. |
| UPF Subgroup 5: Flavored dairy products | Flavored milk, sweetened yogurt, plant-based milks. |
| UPF Subgroup 6: Fats, sauces, ready foods | Margarine, sauces, dressings, nuggets, frozen foods. |
