# Supplementary material for: Associations between Dietary Inflammatory Index, ultra-processed food intake, and clinical outcomes in women with lipedema
Source: Front Nutr. 2026 Jun 30;13:1846293. doi: 10.3389/fnut.2026.1846293 (PMC13364962; doi:10.3389/fnut.2026.1846293)
Supplement: Supplementary file 2 [file Table_2.docx]

# As shown in Table , significant associations were observed between dietary variables and clinical parameters. UPF consumption and DII score were positively associated with inflammatory markers and pain scores (p < 0.05), whereas MedDietScore showed negative correlations with these variables (p < 0.05). Additionally, higher UPF intake and DII scores were associated with lower physical quality of life scores (p < 0.05).

**Table. Correlation Matrix for Dietary, Anthropometric, Inflammatory, Pain, and Quality of Life Variables**

| Variable | 1 | 2 | 3 | 4 | 5 | 6 | 7 | 8 | 9 |
| --- | --- | --- | --- | --- | --- | --- | --- | --- | --- |
| 1. UPF %energy | - |  |  |  |  |  |  |  |  |
| 2. DII score | 0.62** | - |  |  |  |  |  |  |  |
| 3. MedDietScore | –0.48** | –0.51** | - |  |  |  |  |  |  |
| 4. BMI (kg/m²) | 0.29* | 0.33** | –0.25* | - |  |  |  |  |  |
| 5. Lower-limb fat (%) | 0.36** | 0.39** | –0.28* | 0.54** | - |  |  |  |  |
| 6. hs-CRP (mg/L) | 0.41** | 0.52** | –0.22* | 0.46** | 0.49** | - |  |  |  |
| 7. IL-6 (pg/mL) | 0.38** | 0.44** | –0.20* | 0.41** | 0.47** | 0.58** | - |  |  |
| 8. Pain (VAS) | 0.47** | 0.43** | –0.31** | 0.28* | 0.39** | 0.49** | 0.52** | - |  |
| 9. Physical QoL (SF-12 PCS) | –0.33** | –0.29* | 0.44** | –0.22* | –0.27* | –0.35** | –0.31** | –0.46** | - |

Notes: p < 0.05, ** p < 0.01.

# ****Table. Structural Equation Modeling Results: Path Coefficients, Indirect Effects, and Model Fit****

### **A. Standardized Factor Loadings for Latent Variables**

| **Latent Variable** | **Observed Indicator** | **Standardized Loading (λ)** | **p-value** |
| --- | --- | --- | --- |
| **Pro-inflammatory Diet** | UPF %energy | 0.77 | <0.001 |
|  | DII score | 0.82 | <0.001 |
|  | MedDietScore (reverse coded) | 0.69 | <0.001 |
| **Inflammation** | hs-CRP | 0.74 | <0.001 |
|  | IL-6 | 0.71 | <0.001 |
|  | TNF-α | 0.68 | <0.001 |

### **B. Structural Path Coefficients**

| **Path** | **Standardized β** | **95% CI** | **p-value** |
| --- | --- | --- | --- |
| Pro-inflammatory diet → Inflammation | **0.58** | 0.41–0.72 | <0.001 |
| Inflammation → Pain (VAS) | **0.46** | 0.27–0.63 | <0.001 |
| Inflammation → Physical QoL | **–0.39** | –0.58 to –0.17 | 0.001 |
| Pro-inflammatory diet → Pain (direct) | **0.21** | 0.03–0.38 | 0.03 |
| Pro-inflammatory diet → Physical QoL (direct) | **–0.24** | –0.41 to –0.07 | 0.01 |

### **C. Indirect and Total Effects**

| **Effect Type** | **Outcome** | **Standardized β** | **p-value** |
| --- | --- | --- | --- |
| **Indirect effect** | Pro-inflammatory diet → Pain (via inflammation) | **0.27** | 0.004 |
| **Indirect effect** | Pro-inflammatory diet → Physical QoL (via inflammation) | **–0.23** | 0.006 |
| **Total effect** | Pro-inflammatory diet → Pain | **0.48** | <0.001 |
| **Total effect** | Pro-inflammatory diet → Physical QoL | **–0.47** | <0.001 |

### **D. Model Fit Indices**

| **Fit Index** | **Value** | **Interpretation** |
| --- | --- | --- |
| CFI | **0.94** | Good fit |
| TLI | **0.92** | Acceptable–good fit |
| RMSEA | **0.056** (90% CI: 0.034–0.075) | Good fit |
| SRMR | **0.043** | Good fit |
| χ² / df | 1.84 | Acceptable fit |

## **Abbreviations; **UPF:**** Ultra-Processed Food, ****DII:**** Dietary Inflammatory Index, ****QoL:**** Quality of Life, ****VAS:**** Visual Analog Scale, ****CFI:**** Comparative Fit Index, ****TLI:**** Tucker–Lewis Index, ****RMSEA:**** Root Mean Square Error of Approximation, ****SRMR:**** Standardized Root Mean Square Residual, ****CI:**** Confidence Interval.
